# Protein Tyrosine Kinase 7 Regulates EGFR/Akt Signaling Pathway and Correlates With Malignant Progression in Triple-Negative Breast Cancer

**DOI:** 10.3389/fonc.2021.699889

**Published:** 2021-07-22

**Authors:** Nai-Peng Cui, Shu Qiao, Shan Jiang, Jin-Lin Hu, Ting-Ting Wang, Wen-Wen Liu, Yan Qin, Ya-Nan Wang, Li-Shuang Zheng, Jin-Chao Zhang, Yong-Ping Ma, Bao-Ping Chen, Jian-Hong Shi

**Affiliations:** ^1^ Department of Breast Surgery, Hebei Key Laboratory of Cancer Radiotherapy and Chemotherapy, Affiliated Hospital of Hebei University, Baoding, China; ^2^ Institute of Life Science and Green Development, Hebei University, Baoding, China; ^3^ Central Laboratory, Hebei Key Laboratory of Cancer Radiotherapy and Chemotherapy, Affiliated Hospital of Hebei University, Baoding, China; ^4^ Department of Pathology, Affiliated Hospital of Hebei University, Baoding, China; ^5^ College of Chemistry and Environmental Science, Key Laboratory of Analytical Science and Technology of Hebei Province, and MOE Key Laboratory of Medicinal Chemistry and Molecular Diagnostics, Hebei University, Baoding, China; ^6^ Department of Stomatology, Baoding Second Hospital, Baoding, China

**Keywords:** PTK7, triple-negative breast cancer (TNBC), migration, progression, EGFR

## Abstract

**Purpose:**

Triple-negative breast cancer (TNBC), the most aggressive subtype of breast cancer, is associated with high invasiveness, high metastatic occurrence and poor prognosis. Protein tyrosine kinase 7 (PTK7) plays an important role in multiple cancers. However, the role of PTK7 in TNBC has not been well addressed. This study was performed to evaluate the role of PTK7 in the progression of TNBC.

**Methods:**

Correlation of PTK7 expression with clinicopathological parameters was assessed using tissue microarray immunohistochemistry (IHC) staining in 280 patients with breast cancer. PTK7 expression in TNBC (MDA-MB-468, MDA-MB-436 and MDA-MB-231) and non-TNBC (MCF7 and SK-BR-3) breast cancer cell lines were examined using immunoblotting assay. PTK7 correlated genes in invasive breast carcinoma were analyzed using cBioPortal breast cancer datasets including 1,904 patients. PTK7 overexpressed or knockdown TNBC cell lines (MDA-MB-468 and MDA-MB-436) were used to analyze the potential roles of PTK7 in TNBC metastasis and tumor progression. A TNBC tumor bearing mouse model was established to further analyze the role of PTK7 in TNBC tumorigenicity *in vivo*.

**Results:**

PTK7 is highly expressed in breast cancer and correlates with worse prognosis and associates with tumor metastasis and progression in TNBC. Co-expression analysis and gain- or loss-of-function of PTK7 in TNBC cell lines revealed that PTK7 participates in EGFR/Akt signaling regulation and associated with extracellular matrix organization and migration genes in breast cancer, including COL1A1, FN1, WNT5B, MMP11, MMP14 and SDC1. Gain- or loss-of-function experiments of PTK7 suggested that PTK7 promotes proliferation and migration in TNBC cell lines. PTK7 knockdown MDA-MB-468 cell bearing mouse model further demonstrated that PTK7-deficiency inhibits TNBC tumor progression *in vivo*.

**Conclusion:**

This study identified PTK7 as a potential marker of worse prognosis in TNBC and revealed PTK7 promotes TNBC metastasis and progression *via* EGFR/Akt signaling pathway.

## Introduction

Triple-negative breast cancer (TNBC) is the most aggressive subtype of breast cancer characterized by high invasiveness, metastasis and heterogeneous clinical behavior (1–3). Due to lacking expression of estrogen receptor (ER), progesterone receptor (PR), or human epidermal growth factor receptor 2 (HER2), TNBC patients are not sensitive to endocrine therapy or HER2-targeted therapy (4, 5). Resistance to conventional systemic radiotherapy and chemotherapy and high occurrence of post-chemotherapy metastasis make it urgent to develop new TNBC treatment strategies (6–8). Therefore, the importance of understanding the molecular biology of TNBC has gained considerable attention.

Protein tyrosine kinase 7 (PTK7), a member of the receptor tyrosine kinase (RTK) superfamily, is a catalytically inactive RTK that plays a role in multiple cellular processes including polarity and adhesion (9–12). PTK7 interacts with Wnt3a and Wnt8 and acts as an important regulator of both non-canonical and canonical Wnt signaling in multiple developmental contexts (13, 14). PTK7 activates AP-1 and NF-κB signaling and upregulates matrix metalloproteinase-9 (MMP9) which results in increasing invasive properties of esophageal squamous cell carcinoma cells (15). PTK7 binds and activates FGFR1 and increases tumorigenicity (16). Furthermore, PTK7 regulates the activity of kinase insert domain receptor (KDR) and thereby participates in VEGF induced tumor angiogenesis (17).

The expression and function of PTK7 have been investigated in several human cancers, although controversial results have been obtained (18–24). PTK7 is highly expressed and plays an oncogenic role in lung adenocarcinoma (18). PTK7 is overexpressed and contributes to thyroid (19) and cervical (22) cancer progression. A bioinformatics analysis reported that PTK7 is highly expressed in stage I-IV hepatocellular carcinoma (HCC) and considered as an independent prognostic marker for reduced overall survival (21). Another investigation of PTK7 expression in 79 consecutive invasive breast cancer tissues by immunohistochemistry found that PTK7 expression level negatively associates with tumor grade and lymph node metastasis (23). However, Gartner and colleagues found elevated PTK7 mRNA expression level in TNBC cell lines and PTK7 overexpression in metastatic lymph node predicts shorter disease-free survival (DFS) in breast cancer patients (24). The controversy of PTK7 function in breast cancer may be due to its multiple molecular subtypes and heterogeneity.

Although some lines of evidence revealed the important role of PTK7 in tumor progression, the molecular functions of PTK7 in metastasis and motility in TNBC remains elusive. Here we demonstrate that PTK7 were predominantly upregulated in breast cancer tissues. Expression levels of PTK7 predict a poor outcome and an increased risk for cancer metastasis in TNBC patients. Moreover, PTK7 regulates tumor metastasis and collagen fibril organization *via* EGFR-Akt pathway.

## Material and Methods

### Plasmid Constructs and Reagents

Antibodies for PTK7 (25618, 1:1,000) and phosphor-Akt (S473) (4060, 1:1,000) were purchased from Cell Signaling Technology (Danvers, MA, USA). Antibody for β-actin (AC026, 1:20,000) was from Abclonal (Wuhan, Hubei, China). Antibody for Tubulin (10068-1-AP, 1:1,000) was from Proteintech (Chicago, IL, USA). Antibody for EGFR (1114-1, 1:1,000) was from Epitomics (Burlingame, CA, USA). Antibody for phosphor-EGFR (Y1173) (ET1610-4, 1:1,000) was from HuaBio (Hangzhou, Zhejiang, China). Antibody for Akt (B-1, 1:1,000) was from Santa Cruz Biotechnology (Santa Cruz, CA, USA). The human PTK7 expression plasmid was from Addgene (Watertown, MA, USA). LV3 lentiviral vectors encoding shRNAs silencing PTK7 or a nonsilencing control shRNA (shNC) were purchased from GenePharma (Suzhou, Jiangsu, China). The sequences of PTK7 shRNAs: shPTK7#1: 5’-GGATGATGTCACTGGAGAAGA-3’; shPTK7#2: 5’-GGAGGGAGTTGGAGATGTTTG-3’. For gene silencing, 293T cells were transfected with lentiviral vectors together with packaging plasmids and packaged lentiviral particles were prepared and used to infect indicated cells followed by puromycin selection.

### Patients and Tissue Microarray

Two tissue microarrays containing 280 cases of breast cancer tissues collected from 2006 to 2016 with overall survival time (3- to 11-year follow-up, mean follow-up time was 106 months) were purchased from BioChip (Shanghai, China). The breast cancers were divided into the four intrinsic subtypes, Luminal A, Luminal B, HER2(+) and TNBC, based on immunohistochemistry (IHC) results for ER, PR, HER2 and Ki67 provided by BioChip. ER, PR and HER2 positivity was defined using 2018 ASCO/CAP guidelines. ER and PR positivity was defined as ER ≥ 1%, PR ≥ 1%, respectively. For HER2, IHC 3+ or IHC 2+/ISH+ was defined as HER2 positive. ER positive, PR ≥ 20% and Ki67 < 15% was defined as Luminal A. ER positive, PR < 20% and Ki67 > 30% was defined as Luminal B. All the patients provided informed consent. The study was approved by Institutional Review Board of Hebei University Affiliated Hospital. The patient information and histological features were displayed in **Tables 1** and **2**. The analysis of clinicopathological features were based on 280 breast cancer cases or 49 TNBC cases where indicated, excluding a few cases because of missing data.

**Table 1:** Patient characteristics.

Variable	No. of Patients (%)
No. of BC patients	280 (100)
Age: Median [range]	59 [29-88]
Molecular typing	
Luminal A	96 (37.5)
Luminal B	37 (14.5)
HER2(+)	74 (28.9)
TNBC	49 (19.1)
TNM stage	
I	57 (20.4)
II	138 (49.3)
III	81 (28.9)
Lymphatic metastasis	
Negative	143 (51.3)
Positive	136 (48.7)
Distant metastasis	
Negative	280 (100)
Positive	0 (0)
Prognosis	
Survival	208 (74.3)
Death	72 (25.7)

**Table 2:** Molecular subtyping and clinical characteristics.

Variable	Molecular subtyping			
	Luminal A	Luminal B	HER2(+)	TNBC
No. of subtyping patients: n (%)	96 (100)	37 (100)	74 (100)	49 (100)
Age: Median [range]	61 [37-88]	66 [41-88]	57 [33-87]	57 [32-84]
TNM stage: n (%)				
I	21 (22.1)	7 (18.9)	16 (22.2)	12 (25)
II	46 (48.4)	24 (64.9)	33 (45.8)	18 (37.5)
III	28 (29.5)	6 (16.2)	23 (32.0)	18 (37.5)
Lymphatic metastasis: n (%)				
Negative	47 (51.6)	20 (58.8)	36 (50.7)	26 (53.1)
Positive	44 (48.4)	14 (41.2)	35 (49.3)	23 (46.9)
Prognosis: n (%)				
Survival	79 (82.3)	34 (91.9)	52 (70.3)	30 (61.2)
Death	17 (17.7)	3 (8.1)	22 (29.7)	19 (38.8)
PTK7 expression				
IHC score: Mean ± s.d.	5.06 ± 2.42	5.12 ± 2.39	6.20 ± 2.41	7.46 ± 2.68
Low PTK7 level: n (%)	36 (37.5)	13 (35.1)	20 (27.0)	6 (12.2)
Medium PTK7 level: n (%)	41 (42.7)	14 (37.8)	25 (33.8)	12 (24.5)
High PTK7 level: n (%)	19 (19.8)	10 (27.0)	29 (39.2)	31 (63.3)

### IHC Staining

Tissue microarrays were treated with heat-induced antigen-retrieval procedures and IHC staining was performed using the avidin–biotin complex method. The tissue sections were blocked with 10% goat serum and incubated with anti-PTK7 antibody (25618; 1:1,000 dilution; Cell Signaling Technology) at 4°C overnight. Then, the slides were washed three times using PBS followed by biotinylated-secondary antibody incubation for 2 hours at room temperature. The slides were washed three times and incubated with streptavidin/HRP. DAB peroxidase substrate was utilized for visualization. The IHC staining was assessed by two pathologists who were blinded to clinical information. PTK7 IHC score was assessed according to the staining intensity (no staining = 0; weak staining = 1, moderate staining = 2 and strong staining = 3) and the percentage of stained cells (0–4% = 0, 5%–25% = 1, 26%–50% = 2, 51%–75% = 3 and 76%–100% = 4). IHC score = stained cell percentage score × staining intensity score. PTK7 protein expression was divided into low expression (IHC score 0~4), medium expression (IHC score 4~8) and high expression (IHC score 8~12) according to the IHC score.

### PTK7 Gene Expression Profiling

GEPIA: Gene Expression Profiling Interactive Analysis system (http://gepia.cancer-pku.cn/), a newly developed interactive web server for analyzing the RNA sequencing expression data was used to analyze PTK7 expression in breast invasive carcinoma (n = 1,085) and matched normal breast tissues (TCGA normal and GTEx dataset, n = 291). PTK7 expression according to triple-negative status using Breast Cancer Gene-Expression Miner v4.3 system (http://bcgenex.centregauducheau.fr/BC-GEM/). TNBC (n = 572) and non-TNBC breast cancer (n = 6,739) DNA microarray data were selected. For PTK7 genetic alteration analysis in invasive breast carcinoma, cBioPortal for Cancer Genomics (http://www.cbioportal.org/) breast cancer datasets were used which includes 1,904 patients with Agilent microarray data (METABRIC, Nature 2012 & Nat Commun 2016).

### Recurrence-Free Survival (RFS) Assay by Kaplan-Meier Plotter

The prognostic value of PTK7 mRNA expression was evaluated using an online database, Kaplan-Meier Plotter (http://www.kmplot.com/). To analyze RFS of patients with Luminal A, Luminal B, HER2(+) and TNBC subtypes of breast cancer, patients were divided into two groups (high versus low expression) and assessed by a Kaplan-Meier survival plot, with the hazard ratio (HR) with 95% confidence intervals (CIs) and log rank P-value.

### KEGG, GO and PTK7 Correlated-Gene Analysis

PTK7 correlated genes were investigated using breast cancer datasets including 1,904 patients with Agilent microarray data (http://www.cbioportal.org/). Positively- (Spearman’s correlation > 0.3, *P* < 0.01) and negatively- (Spearman’s correlation < -0.3, *P* < 0.01) correlated genes were selected as candidate PTK7 correlated genes. PTK7 correlated genes were analyzed using Kyoto Encyclopedia of Genes and Genomes (KEGG) by DAVID: Functional Annotation Tools (https://david.ncifcrf.gov/tools.jsp) and Gene Ontology (GO) was performed using DAVID: Functional Annotation Tools (https://david.ncifcrf.gov/tools.jsp). Pair-wise gene correlation of PTK7 with EGFR, COL1A1, FN1, WNT5B, MMP11, MMP14 and SDC1 in breast invasive carcinoma were analyzed using GEPIA Correlation Analysis tools (http://gepia.cancer-pku.cn/detail.php?clicktag=correlation).

### Cell Culture

Human TNBC cell lines MDA-MB-436, MDA-MB-468, MDA-MB-231, MCF7 and SK-BR-3 were obtained from Cell Resource Center (IBMS, CAMS/PUMC, Beijing, China). Human embryo kidney 293T cell line was obtained from Cell Resource Center of Shanghai Institutes for Biological Sciences, Chinese Academy of Science, China. MDA-MB-436, MDA-MB-468 and MDA-MB-231 were cultured in RPMI-1640 medium supplemented with 10% FBS, 100 U/mL of penicillin, and 100 μg/mL of streptomycin. HEK293T, MCF7 and SK-BR-3 cells were maintained in Dulbecco’s Modified Eagle’s Medium (DMEM) with 10% FBS, 100 U/mL of penicillin, and 100 μg/mL of streptomycin. All cell lines were cultured in a humidified atmosphere of 5% CO_2_, 95% air at 37°C.

### Gene Silencing

For gene silencing, HEK293T cells were transfected with LV3 lentiviral vectors encoding specific shRNAs targeting PTK7 (shPTK7#1 and shPTK7#2) or control shRNAs (shNC) along with packaging plasmids psPAX2 and pMD2.G. The supernatant was collected at 48 hours after transfection and filtered through a 0.45 μm polysulfone filter for lentiviral particles preparation. MDA-MB-436 and MDA-MB-468 cells were than transduced with the packaged virus and selected by puromycin to establish stable cell lines. Immunoblotting assays were performed to examine the silencing efficiency.

### Immunoblot Assay

Total cell lysates were prepared using RIPA buffer (50 mM Tris-HCl, pH7.4, 150 mM NaCl, 1% NP-40, 0.5% Na-deoxycholate, 1 mM EDTA, 1 mM PMSF, 1 mM DTT, protease inhibitor cocktail). Cell lysates were separated by SDS-PAGE, transferred to PVDF membranes, blocked with 5% non-fat milk and incubated with a specific primary antibody. The membranes were then washed and incubated with HRP-conjugated secondary antibody and visualized by chemiluminescent detection (ECL, Roche Diagnostics, Penzberg, Germany) and exposure to X-ray film (Thermo Fisher Scientific, Waltham, MA, USA). The experiment was repeated independently 3 times.

### Actin Cytoskeleton Staining

Cells were fixed in 4% paraformaldehyde at room temperature for 10 min followed by permeabilization with 0.1% Triton X-100. Cells were incubated with TRITC-tagged phalloidin in the dark at room temperature for 30 min and stained with 4’,6-diamidino-2-phenylindole (DAPI) for 3 min to visualize nuclear. Confocal microscopy was performed with the Confocal Laser Scanning Microscope Systems (FV3000, Olympus, Shinjuku, Japan). The experiment was repeated independently 3 times.

### Cell Proliferation Assays

For MTT assay, 1×10^4^ cells were seeded into 96-well plates and cultured for 0, 24, 48 or 72 hours. Before detection, each well was added with 20 μL MTT reagent (0.5 mg/mL in PBS) followed by an additional 2 hours incubation. The medium was removed and purple-blue MTT formazan precipitate was dissolved in 100 μL DMSO for 10 min at room temperature. The absorbance was measured at 490 nm using a BioTek Epoch Spectrophomometer (BioTek, Winooski, VT, USA). For colony formation, a single-cell suspension was prepared and cells were seeded into a 6-well plate in a concentration at 750 cells/mL and incubated for 2 weeks. Cells were stained with crystal violet and colony formation was photographed under a phase-contrast microscope and colony numbers and diameters were measured. All the experiments were repeated independently 3 times.

### Cell Invasion Assay

Cell invasion assay were performed using a modified Boyden transwell system. The transwell permeable supports chambers (Corning Incorporated, Corning, NY, USA) with 8-μm pore size were pre-coated with 10 mg/L Matrigel overnight at 4°C and 1×10^5^ cells were seeded to the upper chamber of the transwell system and incubated at 37°C for 24 hours. Cells remaining on the upper chamber were mechanically erased with a cotton swab and the cells migrated to the lower surface of the filter were stained with crystal violet and counted under the microscope. The experiment was repeated independently 3 times.

### Tumor Xenograft

Male BALB/c-nu mice at 4-5 weeks old were used to establish TNBC mouse model *in vivo*. All the mice were purchased from the Beijing HFK Bioscience Co., Ltd (Beijing, China) and housed in a specific pathogen-free environment at Hebei University Laboratory Animal Research Center. All experiments were approved by the Animal Research Ethics Committee of the authors’ institution. Briefly, MDA-MB-468 cells (5×10^5^) were injected s.c. into the right mammary fat pad of nude mice. Each group consisted of six mice. The challenged mice were monitored every 2 days for tumor growth. The tumor volume was estimated according to the formula: Volume = 0.5 × *a* × *b* ^2^, where *a* and *b* represent the largest and smallest diameters, respectively. All the mice were sacrificed 61 days after injection and the tumors were weighted, measured and photographed. The experiment was repeated independently 2 times.

### Statistical Analysis

Statistical analysis was performed using GraphPad Prism Software 8.0 (GraphPad Software, San Diego, CA, USA). Two-tailed Student’s *t* tests or one-way ANOVA according to the number of groups compared. *P*-values < 0.05 were considered significant and the level of significance expressed as follows: *, *P* < 0.05; **, *P* < 0.01; ***, *P* < 0.001; ****, *P* < 0.0001.

## Results

### PTK7 Is Highly Expressed and Correlates With Worse Prognosis in TNBC

**Figure 1:**
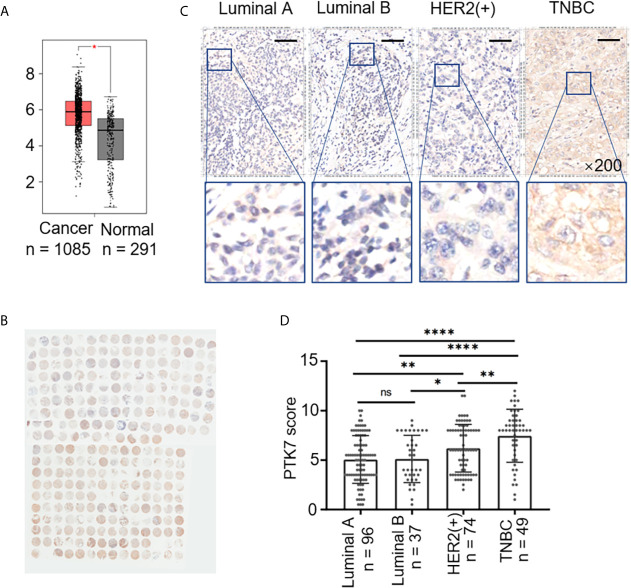
PTK7 expression is upregulated in breast cancer. **(A)** Box plots of PTK7 expression in breast invasive carcinoma (BRCA) using GEPIA: Gene Expression Profiling Interactive Analysis system (http://gepia.cancer-pku.cn/). BRCA tumor (T) and non-tumor (N) TCGA normal and GTEx dataset included 1,085 tumor cases (T) and 291 non-tumor cases (N) was selected to observe the expression of PTK7. **(B)** IHC staining of PTK7 expression in breast cancer tissue microarray. **(C)** Representative images from PTK7 IHC staining in Luminal A, Luminal B, HER2(+) and TNBC subtypes of breast cancer tissues. Magnification, ×200; scale bars, 100 μm. **(D)** Scatter dot plots of PTK7 expression in tumors with different molecular subtypes. Data were analyzed using one-way ANOVA and Tukey’s multiple comparisons test and are shown as mean ± s.d. *P < 0.05, **P < 0.01, ****P < 0.0001. ns, no significance.

To explore the potential role of PTK7 in breast cancer, we analyzed PTK7 expression in breast cancer using an RNA-Seq datasets GEPIA: Gene Expression Profiling Interactive Analysis system (http://gepia.cancer-pku.cn/) and found that PTK7 transcription levels are significantly higher in breast invasive carcinoma (BRCA) tissues (n = 1,085) than that in matched non-tumor tissues (n = 291), suggesting a potential role of PTK7 in breast cancer (**Figure 1A**).

To further investigate the clinical relevance of PTK7, we evaluated breast cancer tissue samples from 280 human subjects (**Table 1**) and performed IHC staining against PTK7 (**Figure 1B**). IHC staining showed that PTK7 was expressed both in the cytosol and the nucleus of breast cancer cells (**Figure 1C**). The samples were divided into four subtypes, Luminal A, Luminal B, HER2(+) and TNBC, based on ER, PR, HER2 and Ki67 expression. Interestingly, PTK7 expression was distinctively higher in TNBC subtype than that in Luminal A, Luminal B and HER2(+) molecular subtypes (**Figures 1C, D**). Next, three TNBC cell lines (MDA-MB-468, MDA-MB-436 and MDA-MB-231), ER(+) breast cancer cell line (MCF7) and HER2(+) breast cancer cell line (SK-BR-3) were used to analyze PTK7 expression and the result showed significantly higher PTK7 levels in TNBC cells than that in MCF7 and SK-BR-3 cells (data not shown).

PTK7 genetic alteration and expression levels were further analyzed using online database in different molecular subtypes of breast cancer. TNBC (n = 572) and non-TNBC breast cancer (n = 6,739) DNA microarray data were selected from Breast Cancer Gene-Expression Miner v4.3 system (http://bcgenex.centregauducheau.fr/BC-GEM/) and PTK7 expressions were higher in TNBC than that in non-TNBC (**Supplementary Figure S1A**). PTK7 genetic alterations in invasive breast carcinoma were analyzed using cBioPortal for Cancer Genomics (http://www.cbioportal.org/) breast cancer datasets and the results revealed that PTK7 genetic amplification exists in 1.6% cases (n = 30) of invasive breast carcinoma patients (n = 1,904), most of which are ER(-), PR(-) and HER2(-) (TNBC, n = 22) (**Supplementary Figure S1B**).

**Figure 2:**
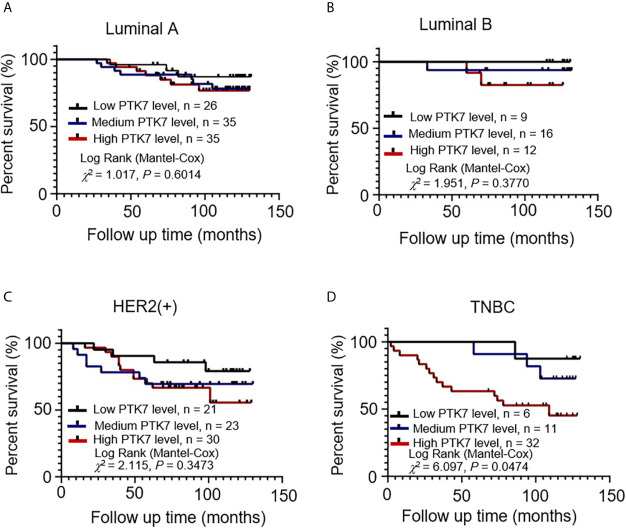
PTK7 upregulation is associated with poor patient survival in TNBC. Breast cancer samples were divided into groups based on PTK7 expression [low expression (IHC score 0~4), medium expression (IHC score 4~8) and high expression (IHC score 8~12)]. Kaplan-Meier overall survival curve analysis and two-sided log-rank tests were performed in Luminal A **(A)**, Luminal B **(B)**, HER2(+) **(C)** and TNBC **(D)** breast cancer molecular subtypes, respectively. Marks on graph lines represent censored samples.

PTK7 expression was qualified as low, medium and high according to IHC score and a follow-up analysis of patient overall survival showed that higher expression of PTK7 in TNBC breast cancer tissue correlated with a worse outcome (**Figure 2D**). However, there was no statistical difference in Luminal A, Luminal B and HER2(+) subtypes (**Figures 2A–C**). Next, we performed RFS analysis using online database Kaplan-Meier Plotter (http://www.kmplot.com/) to assess the effect of PTK7 on breast cancer prognosis. Breast cancer samples were divided into two groups based on PTK7 expression and no significant difference was found Luminal A, Luminal B and HER2 subtypes of breast cancer (**Supplementary Figure S1C**). Interestingly, a significantly worse RFS was found in PTK7 high expressed TNBC (**Supplementary Figure S1C**). These data indicated that PTK7 plays an important role in TNBC and correlated with breast cancer prognosis.

### Elevated PTK7 Is Associated With Tumor Growth and Metastasis in TNBC

**Figure 3:**
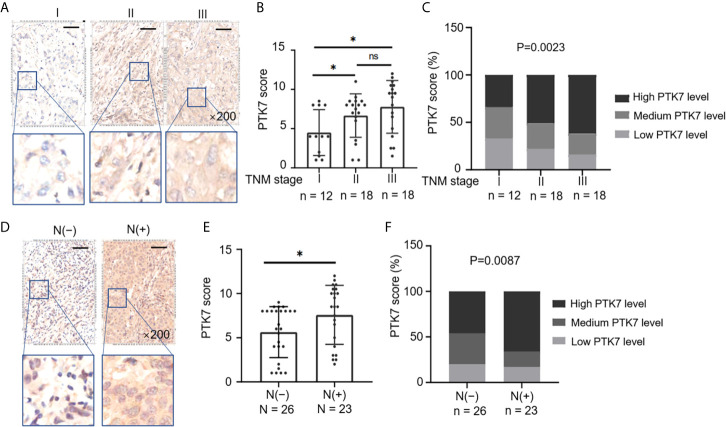
Upregulation of PTK7 is related to metastasis and TNM stage in TNBC. **(A)** TNBC samples from 280 subjects of breast cancer tissue microarray were selected and divided into three groups based on TNM stages (AJCC staging). Representative images of IHC staining of PTK7 expression in the three groups (stage I, II and III) are shown. Magnification, ×200; scale bars, 100 μm. **(B)** Scatter dot plots of PTK7 scores in the three groups described in **(A)**. Data were analyzed using one-way ANOVA and are shown as mean ± s.d. *P < 0.05. **(C)** The percentage of cases in the groups described in **(A)**. Data were analyzed using Pearson’s *χ* ^2^ test. Light grey, low PTK7 level (IHC score 0~4); dark grey, medium PTK7 level (IHC score 5~8); black, high PTK7 level (IHC score 8~12). **(D)** TNBC samples were divided into two groups based on lymph node metastasis. Representative images of PTK7 staining in TNBC with or without lymph node metastasis are shown. Magnification, ×200; scale bars, 100 μm. **(E)** Scatter dot plots of PTK7 scores in the two groups described in **(D)**. Data were analyzed using one-way ANOVA and are shown as mean ± s.d. *P < 0.05. **(F)** The percentage of cases in the groups described in **(D)**. Data were analyzed using Pearson’s *χ* ^2^ test. Light grey, low PTK7 level (IHC score 0~4); dark grey, medium PTK7 level (IHC score 5~8); black, high PTK7 level (IHC score 8~12). ns, no significance.

Next, we selected all the TNBC tissue samples (n = 49) from 280 subjects of breast cancer tissue microarray (**Table 1**) and divided them into groups based on TNM stages and lymph node metastasis. PTK7 expression was significantly higher in TNM II and TNM III groups than that in TNM I group (**Figures 3A, B**). Moreover, elevated PTK7 was observed in TNBC with lymph node metastasis (**Figures 3D, E**). When dividing TNBC tumor samples into groups based on PTK7 IHC staining score, the percentage of high PTK7 expression samples was significantly higher in TNBC with TNM stage III and lymph node metastasis groups (**Figures 3C, F**). These data therefore collectively suggested that PTK7 participates in tumor metastasis in TNBC.

### PTK7 Upregulates EGFR/Akt Signaling Activation

**Figure 4:**
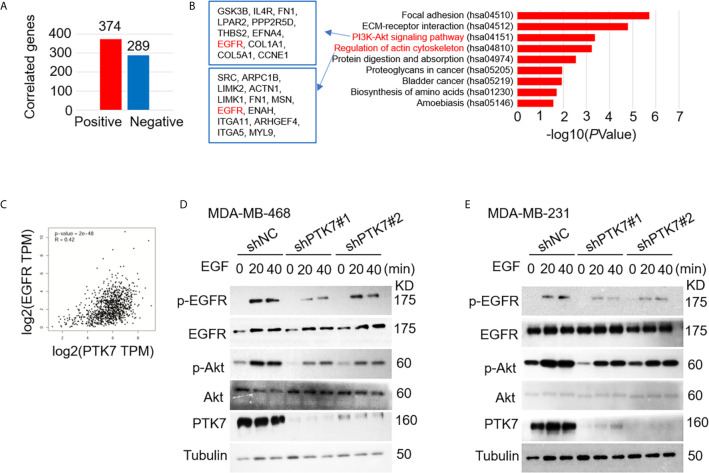
PTK7 regulates EGFR-PI3K-Akt pathway in breast cancer. **(A)** PTK7 co-expression analysis in invasive breast carcinoma using breast cancer datasets including 1,904 patients with Agilent microarray data (http://www.cbioportal.org/) and 374 PTK7 positively- (Spearman’s correlation > 0.3, *P* < 0.01) and 289 PTK7 negative- (Spearman’s correlation < -0.3, *P* < 0.01) correlated genes were selected and used as candidate genes in the following analysis. **(B)** PTK7 positively-correlated genes were analyzed using Kyoto Encyclopedia of Genes and Genomes (KEGG) by DAVID: Functional Annotation Tools (https://david.ncifcrf.gov/tools.jsp). PTK7 positively correlated genes enriched in PI3K-Akt signaling (hsa04151) and Regulation of actin cytoskeleton (hsa04810) pathways are listed in the frames, respectively. **(C)** PTK7 and EGFR pair-wise gene correlation in breast invasive carcinoma were analyzed using GEPIA Correlation Analysis tools (http://gepia.cancer-pku.cn/detail.php?clicktag=correlation). **(D, E)** MDA-MB-468 **(D)** and MDA-MB-231 **(E)** cells were transduced with a non-targeting control shRNA (shNC) or two different PTK7-specific shRNAs (shPTK71 and shPTK7#2). Cells were stimulated with EGF (500 ng/ml) for 0, 20 or 40 minutes and phospho-EGFR and phosphor-Akt levels were evaluated using immunoblotting assay.

We next analyzed PTK7 co-expression genes using breast cancer datasets including 1,904 patients with Agilent microarray data (http://www.cbioportal.org/). As shown in **Figure 4A**, **Supplementary Figure S2** and **Supplementary Table S1**, 374 PTK7 positively-correlated genes (Spearman’s correlation > 0.3, *P* < 0.01) and 289 PTK7-negatively-correlated genes (Spearman’s correlation < -0.3, *P* < 0.01) was found. The functions of PTK7 positively-correlated genes were predicted by the analysis of Kyoto Encyclopedia of Genes and Genomes (KEGG) by DAVID: Functional Annotation Tools (https://david.ncifcrf.gov/tools.jsp) and 9 pathways related to the functions of PTK7 alterations in invasive breast cancer were found (**Figure 4B**, right panel). PI3K/Akt signaling pathway (hsa04151) and actin cytoskeleton regulation (hsa04810) were significantly enriched in PTK7 positively-correlated genes and the associated genes are listed (**Figure 4B**, left panel). To further investigate function of PTK7 in EGFR-PI3K-Akt signaling pathway in breast cancer, we performed PTK7 and EGFR pair-wise gene correlation analysis using GEPIA (**Figure 4C**) and further confirmed EGFR expression positively correlated with PTK7 (R = 0.42, *P* = 2e-48). Then, wild type or PTK7-knockdown TNBC cells (MDA-MB-468 and MDA-MB-231) were stimulated with EGF (500 ng/ml) and phosphor-EGFR and phosphor-Akt levels were significantly lower in PTK7-knockdown cells than that in control cells (**Figures 4D, E**), which suggested that PTK7 regulates EGFR/Akt signaling pathway.

### PTK7 Is Associated With Extracellular Matrix Organization and Cytoskeleton Remodeling in Breast Cancer Cells

**Figure 5:**
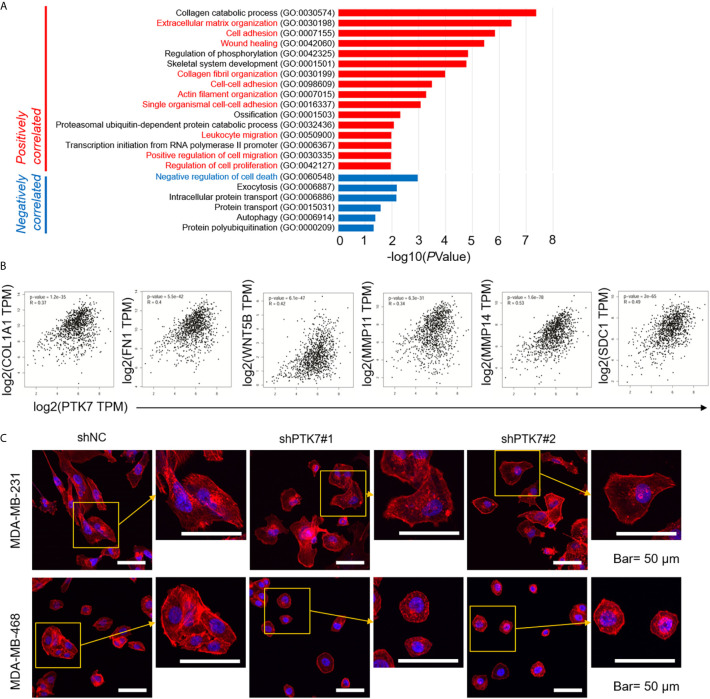
PTK7 associates with extracellular matrix organization and migration in breast cancer cells. **(A)** Gene Ontology (GO) was performed using DAVID: Functional Annotation Tools (https://david.ncifcrf.gov/tools.jsp) and Biological Process (BP) of PTK7 positively- and negatively-correlated genes were shown. **(B)** Pair-wise gene correlation of PTK7 with COL1A1, FN1, WNT5B, MMP11, MMP14 or SDC1 in breast invasive carcinoma were analyzed using GEPIA Correlation Analysis tools (http://gepia.cancer-pku.cn/detail.php?clicktag=correlation). **(C)** MDA-MB-231 and MDA-MB-468 cells were transduced with shNC, shPTK71 or shPTK72. Cells were stained for F-actin with TRITC-phalloidin. Pictures show the TRITC-tagged Phalloidin (red) and DAPI (purple). Presentative images are shown. Magnification, 400×; scale bars, 50 μm.

We further investigated Gene Ontology (GO) using DAVID: Functional Annotation Tools (https://david.ncifcrf.gov/tools.jsp). Biological Process (BP) of PTK7 positively- and negatively-correlated genes showed that 16 biological processes, including extracellular matrix organization (GO:0030198), cell adhesion (GO:0007155), actin filament organization (GO:0007015) and positive regulation of cell migration (GO:0030335) were related to PTK7 positively-correlated genes (**Figure 5A**). To further exam the molecular mechanism, pair-wise gene correlation analysis of PTK7 and key migration associated genes in breast cancer were analyzed using GEPIA correlation analysis tool. As shown in **Figure 5B**, PTK7 expression in breast cancer was significantly positively correlated with COL1A1, FN1, WNT5B, MMP11, MMP14 and SDC1 in breast cancer.

To further identify the potential role of PTK7 in TNBC cytoskeleton remodeling, MDA-MB-231 and MDA-MB-468 cells were transduced with shNC, shPTK7#1 or shPTK7#2. F-actin filaments were stained with phalloidin and the result showed that the actin filaments were recruited into thick and long actin bundles aligned along the long axis in shNC MDA-MB-231 and MDA-MB-468 cells; PTK7-knockdown markedly reduced thick stress fibers (**Figure 5C**).

### PTK7 Promotes Proliferation and Migration in TNBC Cell Lines

**Figure 6:**
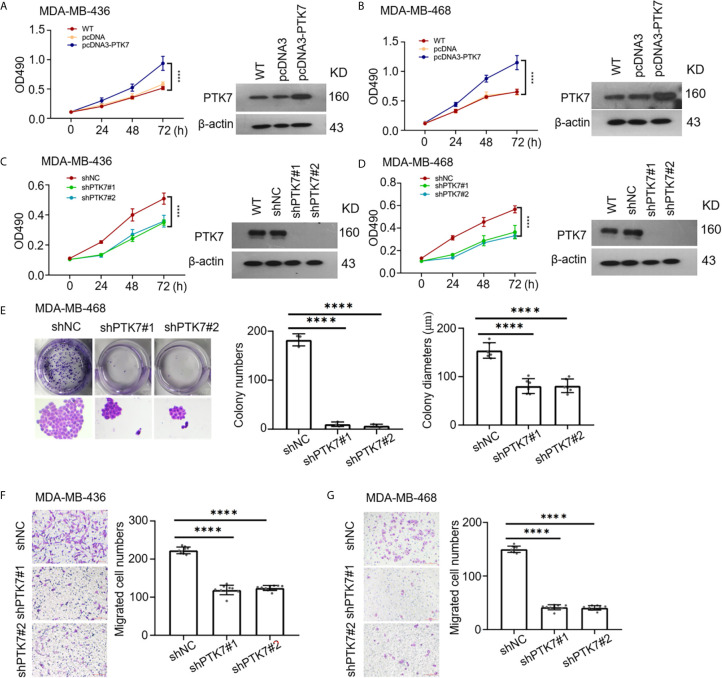
PTK7 participates in cell proliferation and migration in TNBC cell lines. **(A, B)** TNBC cell lines MDA-MB-436 **(A)** and MDA-MB-468 **(B)** were transfected with control vector pcDNA3 or PTK7 expression vector pcDNA3-PTK7 for 48 h and proliferation was evaluated using MTT assay. Data are shown as mean ± s.d. ****P < 0.0001. **(C, D)** MDA-MB-436 **(C)** and MDA-MB-468 **(D)** were transduced with a non-targeting control shRNA (shNC) or two different PTK7-specific shRNAs (shPTK71 and shPTK7#2). Cell proliferation was evaluated using MTT assay. Data are shown as mean ± s.d. ****P < 0.0001. **(E)** Colony formation assay was performed to determine proliferation in shNC-, shPTK7#1- or shPTK7#2-transduced MDA-MB-468 cells. Presentative images are shown (left) and colony numbers and colony diameters were shown as mean ± s.d. Magnification, ×100; ****P < 0.0001. **(F, G)** Transwell migration assay using Boyden chamber in shNC-, shPTK7#1- or shPTK7#2-transduced MDA-MB-436 **(F)** and MDA-MB-468 **(G)** cells was performed and photographed under a light microscope. Presentative images are shown (left) and migrated cells were counted and shown as mean ± s.d. Magnification, ×100; ****P < 0.0001.

To identify the consequences of PTK7 in TNBC progression, human PTK7 overexpression or knockdown TNBC cell lines MDA-MB-436 and MDA-MB-468 were used and MTT cell proliferation assay were performed. As expected, overexpression of PTK7 in MDA-MB-436 and MDA-MB-468 cells significantly promotes proliferation activity (**Figures 6A, B**) and knockdown of PTK7 resulted in a decrease of cell growth (**Figures 6C, D**). Colony formation assay showed that both the colony numbers and colony diameters significantly decreased in PTK7-knockdown cells (**Figure 6E**). Matrigel pre-coated Boyden chamber was then used to analyze the roles of PTK7 in TNBC cell migration and invasion. Knockdown of PTK7 in MDA-MB-436 and MDA-MB-468 cells exhibited decreased migration ability (**Figures 6F, G**), and overexpression of PTK7 promoted transwell migration in TNBCs (**Supplementary Figure S3**).

### PTK7-Deficiency Inhibits TNBC Tumor Growth *In Vivo*

**Figure 7:**
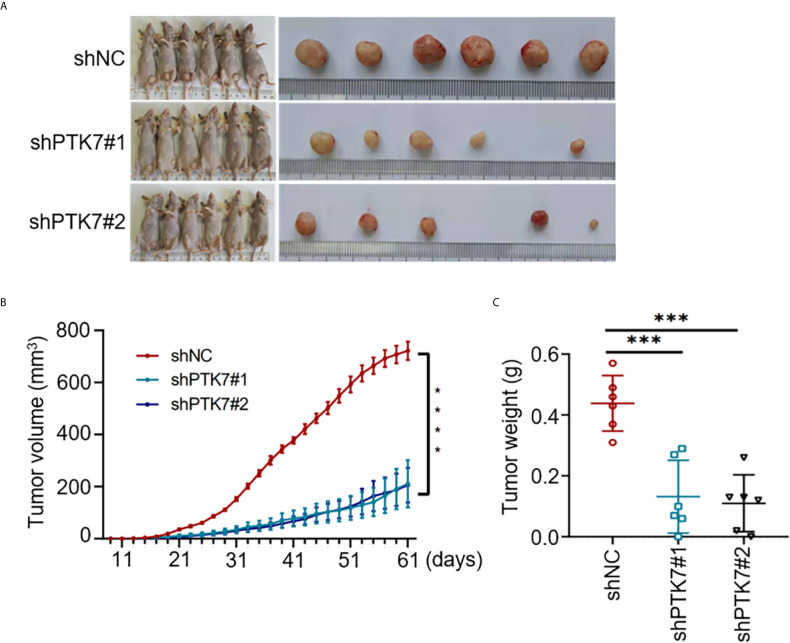
PTK7 promotes in *vivo* TNBC tumorigenicity. **(A)** Nude mice were injected into the mammary fat pad with shNC-, shPTK7#1- or shPTK7#2-transduced MDA-MB-468 cells at 5×10^5^ cells per site. Mice were sacrificed and tumors were photographed 61 days after subcutaneous injection. **(B)** Tumor volume was monitored every 2 days after injection and tumor growth curve is shown. Tumor volume was calculated by the formula: V = 1/2×*a* (length)×*b* ^2^ (width). **(C)** The harvest tumor weight was shown as mean ± s.d. ***P < 0.001; ****P < 0.0001.

To further analyze the role of PTK7 in TNBC tumorigenicity *in vivo*, shNC, shPTK7#1 and shPTK7#2 stable transduced MDA-MB-468 cells were used to establish TNBC tumor bearing mouse model. The challenged mice were monitored every two days and sacrificed at day 61 after injection (**Figure 7A**). PTK7 knockdown dramatically inhibited tumor growth (**Figures 7B, C**). These results suggested that PTK7 is required for TNBC progression *in vivo*.

## Discussion

RTKs, a protein kinase family transducing extracellular signals across the cell membrane, were known to be grouped into 20 subfamilies and play pivotal roles in diverse cellular activities including growth, differentiation, motility, and death (25–28). Many RTKs are involved in oncogenesis (29, 30). PTK7 is a particular member of the RTK family that lacks detectable catalytic tyrosine kinase activity. Although PTK7 plays a role in multiple cellular processes during tumor progression, the definite role of PTK7 in breast cancer progression remains unclear.

A recent meta-analysis of the prognostic value of PTK7 expression in human malignancies revealed that higher expression of PTK7 significantly indicates worse prognosis in human malignancies in 11 studies published with a total sample size of 2431 participants (31). The expression and function of PTK7 in breast cancer have been well investigated, however, controversial results were obtained. Several studies suggested that PTK7 is highly expressed in TNBC cell lines and associates with resistance to anthracycline-based chemotherapy in TNBC (32). PTK7 expression in breast cancer predicts poor prognosis (24). Recent evidence including 79 consecutive invasive breast cancer tissues demonstrated that PTK7 expression is negatively associated with tumor grade and lymph node metastasis and may serve as a tumor suppressor in breast cancer (23).

To reveal the clinical relevance of PTK7 in breast cancer, in the present study, we evaluated breast cancer tissue samples from 280 human subjects and performed tissue microarray IHC staining against PTK7. There was no significant associate of PTK7 expression with TNM stages from totally 280 breast cancer tissues. Interestingly, either correlations of PTK7 expression with clinicopathological parameters by tissue microarray IHC staining or online RFS analysis by Kaplan-Meier Plotter (http://www.kmplot.com/) demonstrated that PTK7 expression extraordinarily correlates with worse prognosis in ER/PR/HER2-negative (TNBC) breast cancer, which suggested a special relationship of PTK7 expression with worse prognosis in TNBC. The function of PTK7 in breast cancer exhibits heterogeneity in multiple molecular subtypes may due to different cell context and intracellular signaling mechanisms.

Compared with Luminal A, Luminal B and HER2(+) breast cancer subtypes, patients with TNBC were always recognized to have the worst overall survival data due to its rapid progression, high probabilities of early recurrence, and distant metastasis resistant to standard treatment (33). According to the present data, TNBC with high PTK7 expression level predicts worse outcome. KEGG analysis and PTK7 gain- or loss-of-function TNBC cell lines revealed that PTK7 regulates EGFR/Akt signaling pathway. GO assay further demonstrated PTK7 participates in extracellular matrix organization and migration in TNBC cells. A recent study revealed that PTK7 expression is associated with EGFR mutations and plays an oncogenic role in lung adenocarcinomas (18). The role of PTK7-targeted antibody-drug conjugate has been investigated in several solid tumors, including TNBC, and exhibits potential therapeutic activity (34–36). In addition, our present data demonstrated that loss of PTK7 expression in TNBC cells results in a downregulated EGFR/Akt signaling and reduced tumor growth in MBA-MD-468 TNBC cancer xenografts. These findings may have significant implicants for the treatment of TNBC *via* targeting PTK7.

Taken together, this study identified PTK7 as a potential marker of worse prognosis in TNBC. PTK7 promotes extracellular matrix organization and migration *via* EGFR/PI3K/Akt signaling pathway in TNBC. Strategies targeting PTK7 may inform the development of novel therapies to fight against TNBC. To further define the independent predictive role and targeted therapy strategy of PTK7 in TNBC, a larger sample of patients with TNBC treatment information should be investigated.

## Data Availability Statement

Publicly available datasets were analyzed in this study. This data can be found here: GEPIA: Gene Expression Profiling Interactive Analysis system (http://gepia.cancer-pku.cn/) cBioPortal for Cancer Genomics (http://www.cbioportal.org/) Kaplan-Meier Plotter (http://www.kmplot.com/) DAVID: Functional Annotation Tools (https://david.ncifcrf.gov/tools.jsp).

## Ethics Statement

The studies involving human participants were reviewed and approved by Ethics Committee of Affiliated Hospital of Hebei University. The patients/participants provided their written informed consent to participate in this study. The animal study was reviewed and approved by Animal Welfare and Ethical Committee of Hebei University.

## Author Contributions

N-PC, SQ, SJ, and J-HS designed and carried out the experiments. SQ and SJ analyzed the data. N-PC, SQ, YQ, Y-NW, and L-SZ performed immunohistochemical staining, tumor xenograft. SJ, J-LH, T-TW, and W-WL performed cell culture, gene silencing, immunoblotting, actin cytoskeleton staining, cell proliferation assays, cell proliferation and invasion assays. J-HS, N-PC, SQ, SJ, and Y-NW analyzed and interpreted the data. J-HS, J-CZ, Y-PM, and B-PC provided supervision and guidance. J-HS, Y-PM, and B-PC wrote the manuscript. All authors contributed to the article and approved the submitted version.

## Funding

This work was supported by the Natural Science Foundation of Hebei Province [grant number H2019201259, C2020201052, H2020201295], the National Natural Science Foundation of China [grant Number 31971304], Hebei Province Foundation for the Returned Overseas Scholars [grant Number C20200305], Government Foundation of Clinical Medicine Talents Training Program of Hebei Province [grant number 361007], Medical Scientific Research Project of Hebei Province [grant number 20200170], Medical Science Foundation of Hebei University [grant number 2020A09] and Hebei University graduate innovation funding project [grant number hbu2020ss081].

## Conflict of Interest

The authors declare that the research was conducted in the absence of any commercial or financial relationships that could be construed as a potential conflict of interest.

## Abbreviations

BP, Biological Process; ER, estrogen receptor; GO, Gene Ontology; HER2, human epidermal growth factor receptor 2; IHC, Immunohistochemistry; KDR, kinase insert domain receptor; KEGG, Kyoto Encyclopedia of Genes and Genomes; MMP9, matrix metalloproteinase-9; PR, progesterone receptor; PTK7, protein tyrosine kinase 7; RFS, recurrence-free survival; RPTK, receptor protein tyrosine kinase; TNBC, triple-negative breast cancer.
