# Supplementary material for: Protein Tyrosine Kinase 7 Regulates EGFR/Akt Signaling Pathway and Correlates With Malignant Progression in Triple-Negative Breast Cancer
Source: Front Oncol. 2021 Jul 22;11:699889. doi: 10.3389/fonc.2021.699889 (PMC8339706; doi:10.3389/fonc.2021.699889)
Supplement: Supplementary file 1 [file DataSheet_1.pdf]

Supplementary Material

Supplementary Figures

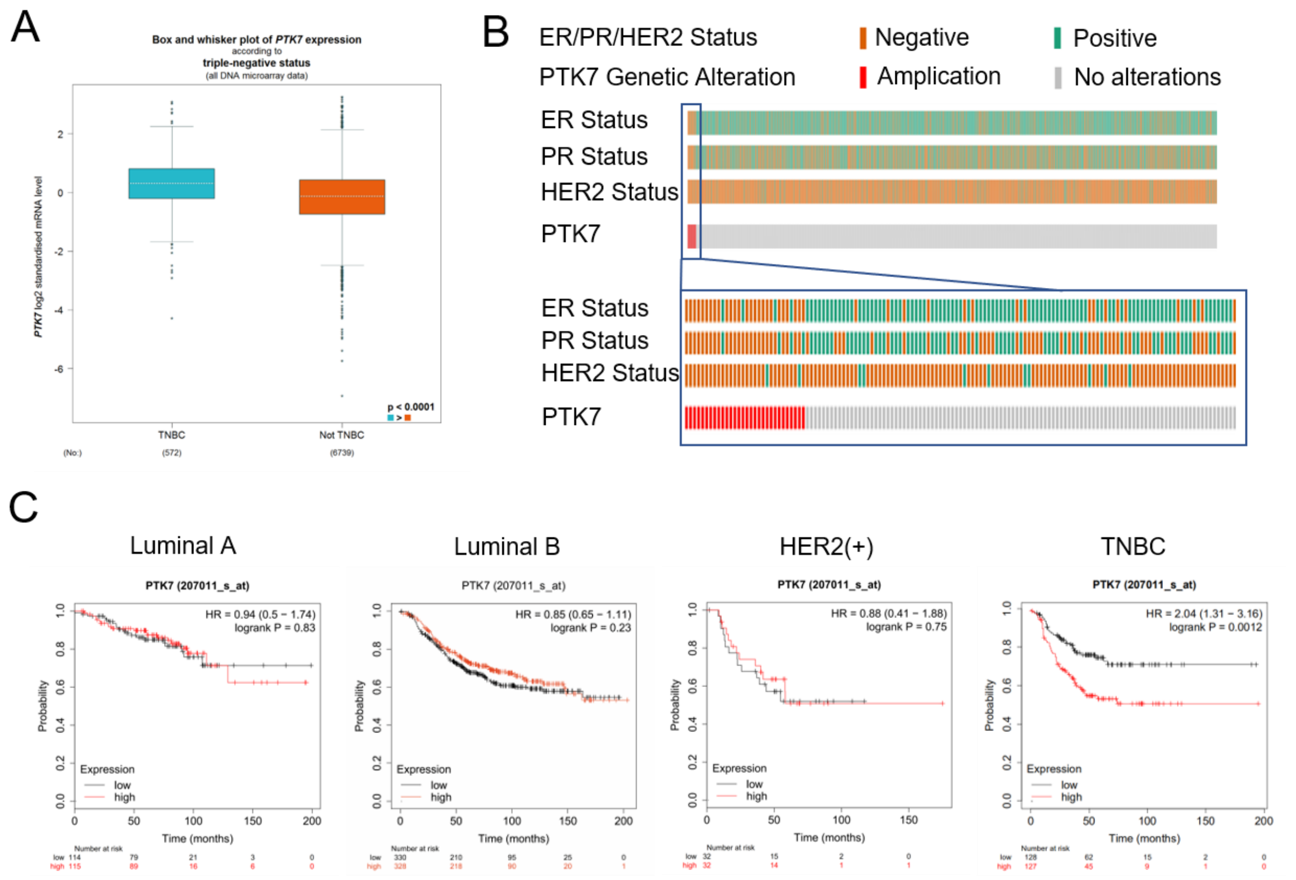

Supplementary Figure S1. PTK7 correlates with a worse prognosis in TNBC.

(A) Box plots of PTK7 expression according to triple-negative status using Breast Cancer Gene-Expression Miner v4.3 system (http://bcgenex.centregauducheau.fr/BC-GEM/). TNBC (n=572) and non-TNBC breast cancer (n=6,739) DNA microarray data were selected.
(B) PTK7 genetic alteration analysis in invasive breast carcinoma using cBioPortal for Cancer Genomics (http://www.cbioportal.org/) breast cancer datasets including 1,904 patients with Agilent microarray data (METABRIC, Nature 2012 & Nat Commun 2016).
(C) Recurrence-free survival (RFS) analysis was performed using Kaplan-Meier Plotter (http://www.kmplot.com/) to assess the effect of PTK7 mRNA expression on survival in Luminal A, Luminal B, HER2(+) and TNBC subtypes of breast cancer. Patients were split into two groups according to PTK7 mRNA median expression levels and the cut off values of Luminal A, Luminal B, HER2(+) and TNBC were 443, 495, 617 and 725, respectively.

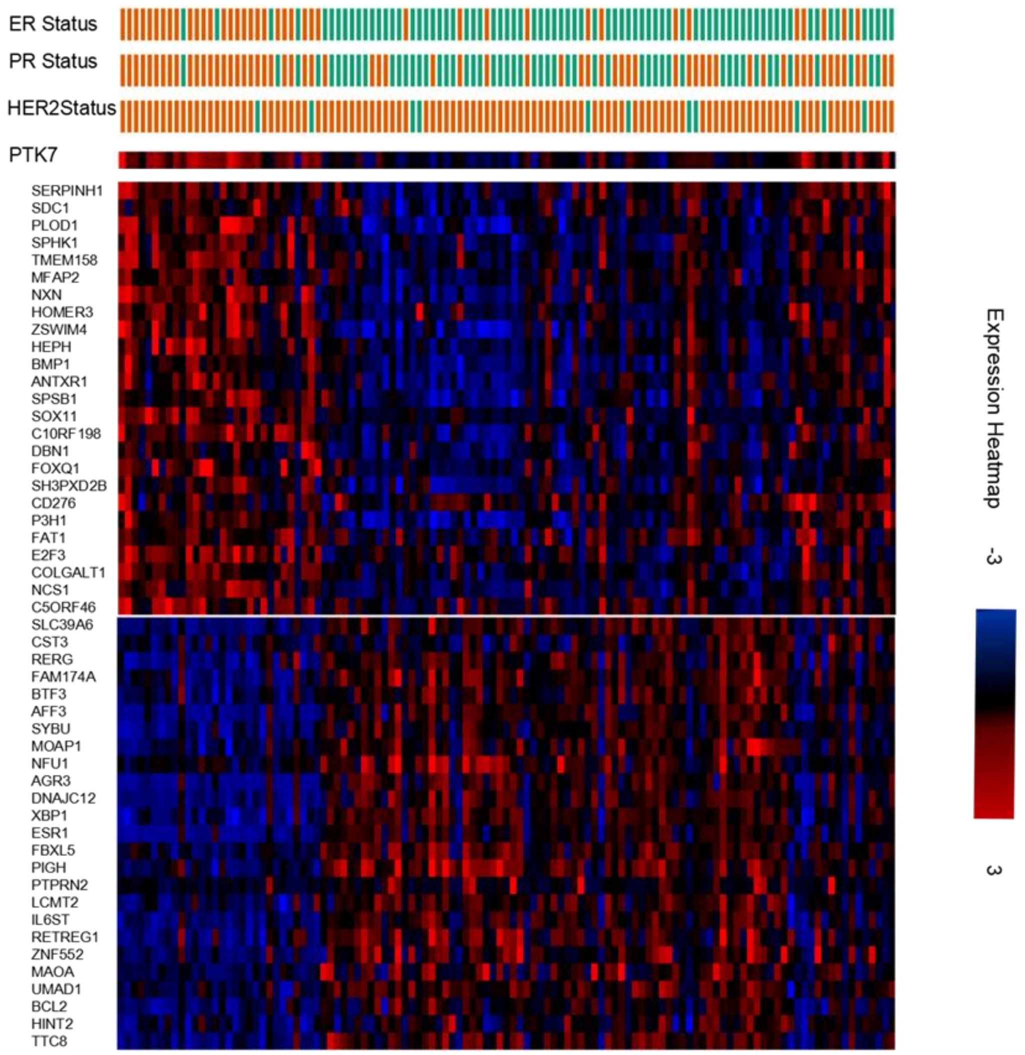

Supplementary Figure S2. PTK7 co-expression genes in invasive breast carcinoma. PTK7 co-expression analysis in invasive breast carcinoma using cBioPortal for Cancer Genomics (http://www.cbioportal.org/) using Agilent microarray data (METABRIC, Nature 2012 & Nat Commun 2016). Some of the positively and negatively correlated genes are shown.

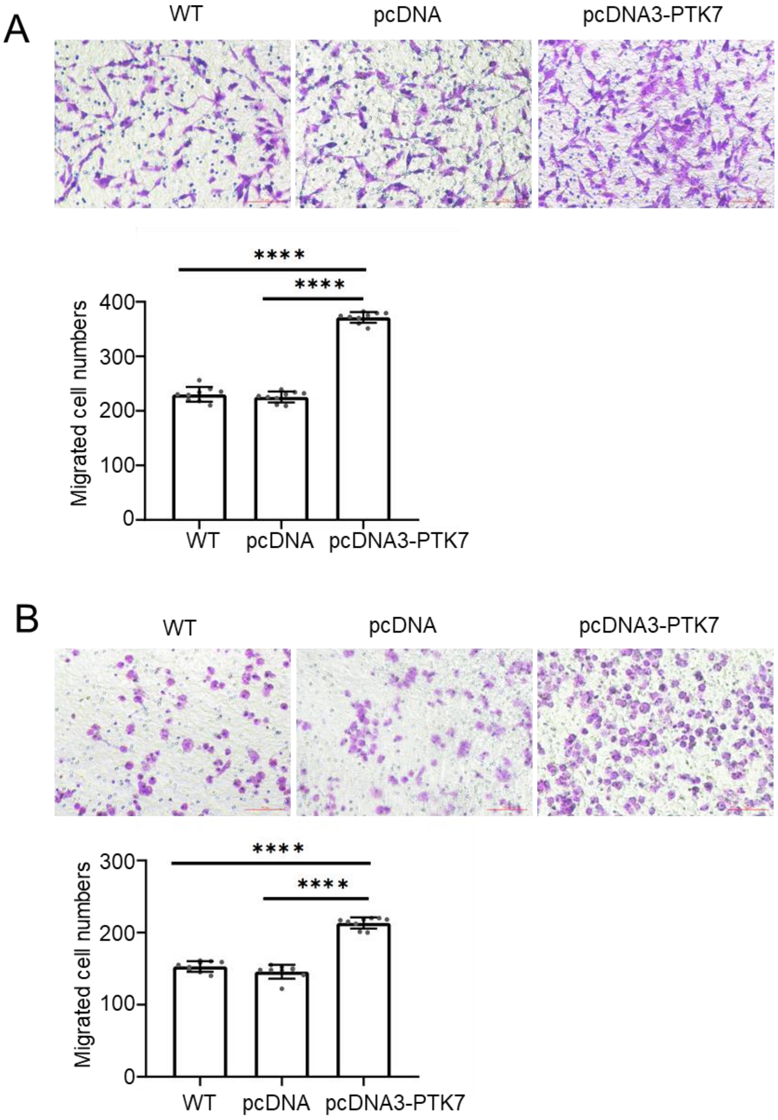

Supplementary Figure S3. Transwell migration assay in PTK7-overexpressed TNBC cell lines. (A and B) Transwell migration assay using Boyden chamber in WT, pcDNA3 or pcDNA3-PTK7 transfected MDA-MB-436 (A) and MDA-MB-468 (B) cells was performed and photographed under a light microscope. Upper, representative images are shown (Magnification, ×100). Lower, migrated cells were counted and shown as mean ± s.d. ****, $P < 0.0001$.
