# Supplementary figures and images for: Protein Tyrosine Kinase 7 Regulates EGFR/Akt Signaling Pathway and Correlates With Malignant Progression in Triple-Negative Breast Cancer
Source: Front Oncol. 2021 Jul 22;11:699889. doi: 10.3389/fonc.2021.699889 (PMC8339706; doi:10.3389/fonc.2021.699889)

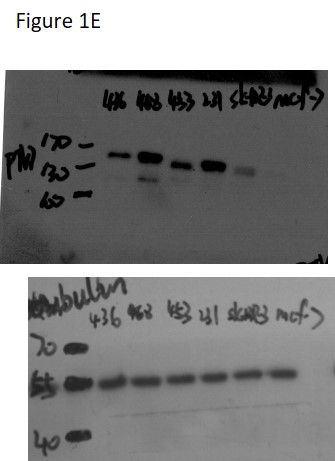

Supplement: Supplementary file 2 [file Image_1.jpeg]

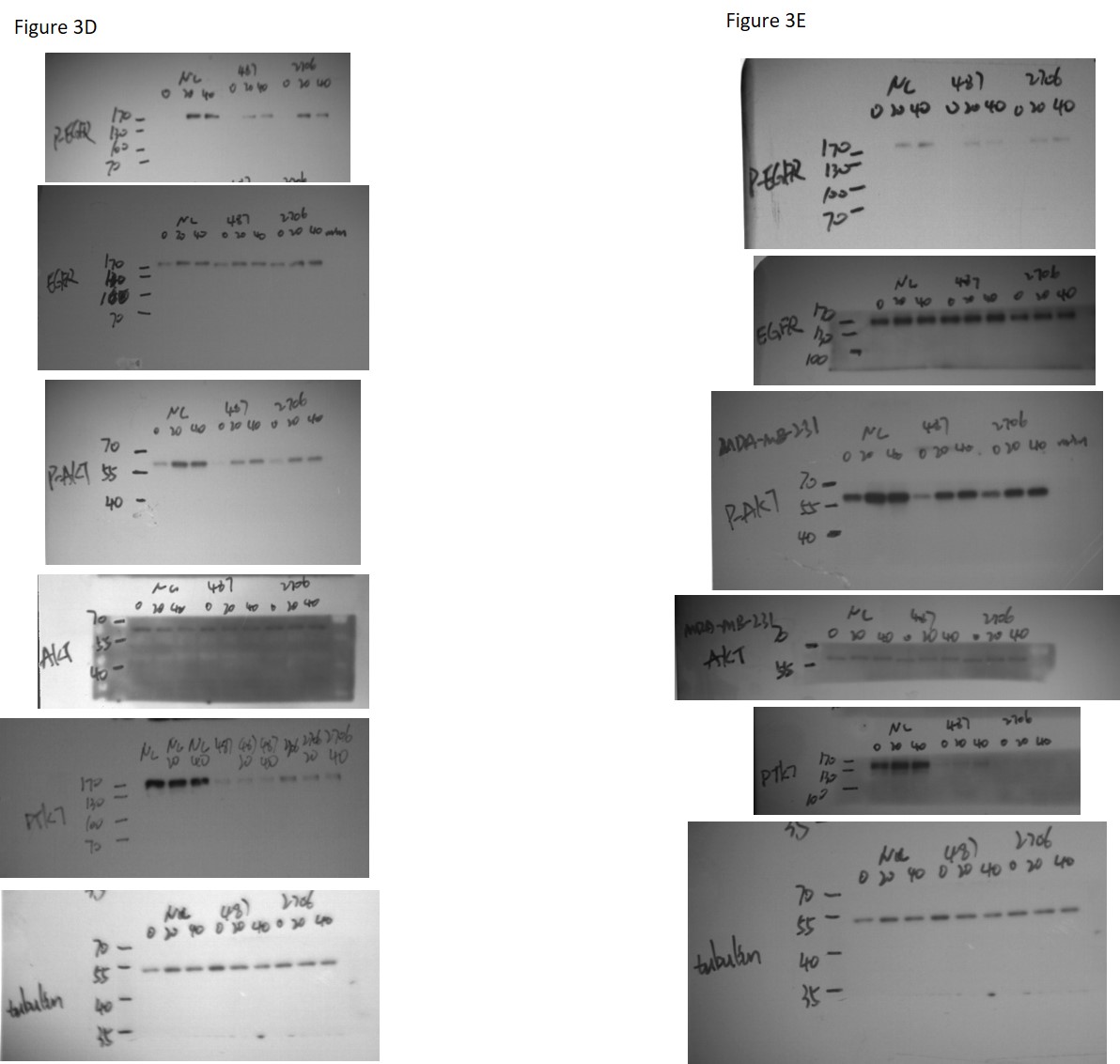

Supplement: Supplementary file 3 [file Image_2.jpeg]

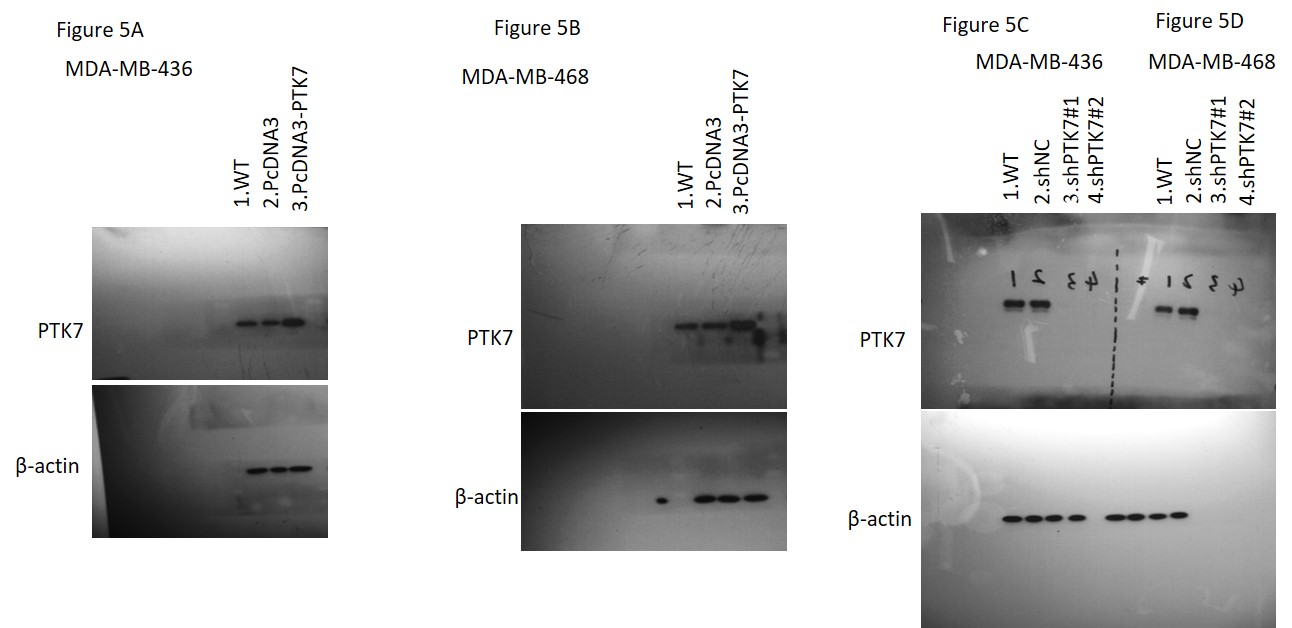

Supplement: Supplementary file 4 [file Image_3.jpeg]
